# Osteogenic Potential of Graphene in Bone Tissue Engineering Scaffolds

**DOI:** 10.3390/ma11081430

**Published:** 2018-08-14

**Authors:** Somasundaram Prasadh, Santhosh Suresh, Raymond Wong

**Affiliations:** Faculty of Dentistry, National University of Singapore, 1 Lower Kent Ridge Road, Singapore 119083, Singapore; e0204949@u.nus.edu (S.P.); santhosh.suresh@u.nus.edu (S.S.)

**Keywords:** graphene, bone tissue engineering, bone scaffold, stem cells, biomaterials

## Abstract

Scaffolds are physical substrates for cell attachments, proliferation, and differentiation, ultimately leading to tissue regeneration. Current literature validates tissue engineering as an emerging tool for bone regeneration. Three-dimensionally printed natural and synthetic biomaterials have been traditionally used for tissue engineering. In recent times, graphene and its derivatives are potentially employed for constructing bone tissue engineering scaffolds because of their osteogenic and regenerative properties. Graphene is a synthetic atomic layer of graphite with SP2 bonded carbon atoms that are arranged in a honeycomb lattice structure. Graphene can be combined with natural and synthetic biomaterials to enhance the osteogenic potential and mechanical strength of tissue engineering scaffolds. The objective of this review is to focus on the most recent studies that attempted to explore the salient features of graphene and its derivatives. Perhaps, a thorough understanding of the material science can potentiate researchers to use this novel substitute to enhance the osteogenic and biological properties of scaffold materials that are routinely used for bone tissue engineering.

## 1. Introduction

Tissue engineering utilizes the concept of scaffolding to regenerate the bone defects. The technique for addressing these defects that involves the reconstruction or regeneration of bone always desires a temporary porous scaffold. Bone defects that are caused by trauma, congenital malformations, or due to cancerous lesions can best be therapeutically managed by bone tissue engineering scaffolds. The ultimate aim of modern tissue engineering is to replace the traditional medical procedures involving the repair and regeneration of tissues with three-dimensional scaffolds. These scaffolds have a porous surface that serve as a medium, over which cells can grow rapidly and develop into tissues that are viable. The scaffolds with unique morphology and structural architecture encourage the cells to regenerate bone tissues of extra cellular matrix [1,2]. Ideally, scaffolds provide the anatomical support for the multiplying cells to repair the damaged tissues and in the process, undergo gradual degeneration and resorb without harming the resulting tissues. The scaffolds should be porous, biocompatible, biodegradable, and designed accordingly to satisfy the required needs to regenerate or repair the tissues [3,4,5]. The architecture and composition of the scaffolds are such that, it helps in cell attachments, proliferation and differentiation of the surrounding host cells into the defect area on its surface [6,7,8]. The scaffold materials are classified into natural and synthetic biomaterials. The pore architecture and design of the scaffolds influence their mechanical strength. The square design architecture of the scaffold is said to have high strength when compared to other scaffold designs [9]. Factors such as design, micro-architecture, material property, and strength are essential in stabilizing the surrounding microenvironment and the cells adhering to it [10,11,12]. Synthetic scaffold materials have several advantages over the natural materials in terms of high mechanical strength, biocompatibility, biodegradability, and cost effectiveness [13,14,15]. Biomaterials, like glasses and ceramics, are being used as scaffold materials for bone tissue engineering [16].

The polymeric scaffolds have excellent biocompatibility and biodegradability, but lack the ability to withstand the forces acting upon. In order to improve their mechanical strength, many researchers have incorporated graphene derivatives within the polymers. Besides enhancing the physical properties, the graphene derivatives also promote cell proliferation and differentiation, owing to their excellent biocompatibility under limited concentrations.

Graphene is a single layer of aromatic carbon atoms that are arranged in a two-dimensional lattice [17,18]. Research on graphene and its derivatives have gained immense popularity among industries as well as in academia due to its fascinating biomechanical properties. The global interest in this “wonder material” is still growing, especially in the field of bone tissue engineering and biomedical application. Besides, graphene-based materials have also been used widely in the field of biomedicine and drug delivery. Because of its excellent biocompatibility under recommended concentrations, graphene and its derivatives have shown promising results in regulating cell behavior, loading, and releasing of drug genes. Additionally, it is also believed that they help in the differentiation of cells by modifying the surface of the scaffold materials. Graphene improves the adhesion, growth, proliferation, and differentiation of osteoblast [19]. They also possess unique physio-chemical and mechanical properties, which allow for them to be used with broad range devices and in the form of scaffold materials to enhance the proliferation of stem cells for bone regeneration. In this article, we will be emphasizing on the osteogenic influences of graphene derivatives when combined with different biomaterials for utilization in bone tissue engineering scaffolds.

## 2. Graphene Derivatives

**Figure 1:**
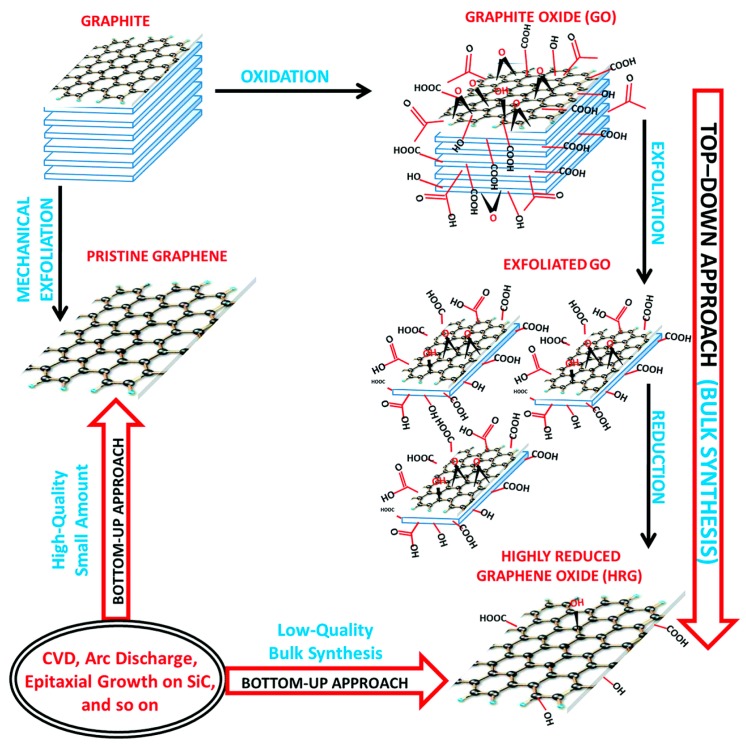
Graphene derivatives, graphene oxide and reduced graphene oxide [29] (copyright 2015, Journal of Materials Chemistry—A).

Graphene is a synthetic atomic layer of graphite with SP^2^ bonded carbon atoms arranged in honeycomb lattice structure. Boehm et al. described graphene in 1986 [20]. In 2004, Geim and Novoselov isolated and identified graphene [21,22]. Graphene remained a popular material that was researched extensively in the past decade for its remarkable electro-conductivity. The graphene and its derivatives have also been noted as components of biosensors or to remotely control cell substrate interfaces [23,24,25,26,27,28]. The derivatives of graphene, (graphene oxide and reduced graphene oxide) have many functional groups on their surface and have unique properties, like graphene (Figure 1). Graphene oxide obtained by the oxidation of graphene, [29] spans the modern trends in bone regeneration [30] because of its unique physical, chemical, mechanical properties, and good biocompatibility [31,32].

Graphene oxide, aids in bone regeneration by enhancing the osteogenic differentiation of progenitor cells. This is accomplished by hydrophobic and electrostatic interactions with the proteins of the microenvironment [31,33,34]. Graphene oxide differs from graphene in that it forms a uniform and stable suspension in water, whereas graphene tends to from aggregates. Uniform stable suspension of graphene oxide helps to infiltrate the porous scaffolds, thereby modifying the surfaces of pore wall [35]. This unique characteristic of graphene oxide makes it the material of choice in bone tissue engineering. The graphene oxide can further be reduced to form the reduced graphene oxide. The graphene oxide has many oxygen containing functional groups on their surface, which facilitates the interaction with cells and biomolecules. Like the graphene oxide, the reduced graphene oxide also contains functional groups on their surface and has a unique electrical property when compared to graphene oxide. Indeed, graphene-based materials have proven to enhance cell growth, cell differentiation, and cell proliferation [36,37].

## 3. Natural vs. Synthetic Biomaterials

Various biomaterials have been researched as scaffold materials for bone tissue engineering. Biocompatibility, biodegradability, and sufficient mechanical strength to withstand the forces acting upon are the three key factors to be considered for the selection of a scaffold [38]. Depending on their use and clinical application, the scaffolds can be classified into synthetic or natural, biodegradable or non-biodegradable, and rigid/non-rigid scaffolds [39].

### 3.1. Natural Biomaterials

Natural biomaterials that are used in bone tissue regeneration include proteins like silk, collagen, gelatin, fibrin, fibrinogen, elastin, keratin, actin, and myosin. On the other hand, polysaccharides, such as chitosan, hyaluronic acid, alginate, agarose, cellulose, amylose, dextran, chitin, and glycosaminoglycan’s; and, polynucleotides, like DNA, RNA, chitosan, hyaluronic acid, alginate, and agarose are also being utilized [40]. The aforementioned proteins and polysaccharides are routinely used with graphene in the field of tissue engineering and biomedical application [41]. The blending of graphene derivatives with these natural polymers enhances the biological properties of the scaffolds. The most researched polysaccharides are chitosan, cellulose, and alginate, whereas the protein-based polymers frequently studied include silk, collagen, and gelatin [42].

#### Natural Biomaterials/Polysaccharides/Graphene Scaffolds in Bone Tissue Engineering

Nishida et al. [43] coated collagen scaffolds with different concentration of graphene oxide and evaluated the bioactivity, cell proliferation, and differentiation both in vivo and in vitro. The results of subcutaneous implant tests in rats showed increased cell growth in low concentrations of graphene oxide. Adverse biological effects where seen in higher concentrations of graphene oxide. Consequently, scaffolds that are modified with a suitable concentration of graphene oxide are useful as a bioactive material for tissue engineering [43]. Kang et al. [44] compared the osteogenic potential of Graphene oxide flakes/collagen scaffolds against collagen scaffolds that served as the control group. qRT-PCR analysis was used to compare the mRNA expression of osteogenic markers, alkaline phosphatase (ALP) and Osteopontin (OP), in human mesenchymal stem cells (hMSCs) cultured on the four types of scaffolds. The mRNA expression of ALP was 3.9 ± 0.2-fold and 4.3 ± 0.3-fold higher for the graphene oxide-collagen group compared with the collagen group at 2 and 3 weeks, respectively. Further, the graphene oxide-collagen group showed significantly enhanced mRNA expression levels of Osteopontin (OP), which was 6.5 ± 1.1 and 7.7 ± 0.6-fold higher than the collagen group at two weeks and three weeks, respectively. Immunocytochemical staining of RUNX2 was performed to observe the osteogenic differentiation of hMSCs on both collagen and graphene oxide-collagen scaffolds. The hMSCs cultured on graphene oxide (GO)-collagen scaffolds showed more enhanced expression of RUNX2 compared to those on collagen scaffolds. The results demonstrated that the graphene oxide/collagen scaffolds showed more osteogenic differentiation of human mesenchymal stem cells when compared to the control collagen scaffolds [44]. 

Another study showed that the addition of graphene oxide or reduced graphene oxide to collagen scaffolds increased the tissue compatibility and bioactivity. In vivo studies showed that reduced graphene oxide is more bioactive than graphene oxide when coated on collagen scaffolds [45]. Dinescu et al. [46] produced a three-dimensionally printed chitosan and graphene oxide scaffolds by using 0.5% and 3% graphene oxide and found that addition of graphene oxide increased the proliferation of murine pre-osteoblast cells and metabolic activity [46]. Three-dimensionally printed graphene oxide and chitosan scaffolds promoted bone regeneration in calvarial defects of mouse. They observed an increase in the osteogenic activity and repair of the critical size defect. The addition of graphene oxide with chitosan showed increase in Alkaline phosphatase activity, expression of bone morphogenic protein and RUNX-2 factor [47]. The pendant hydroxyl group and high polarity of graphene oxide were shown to enhance the cell attachment on the scaffolds printed with chitosan. The negative charge of graphene oxide and its polarity allows for the van der Waals forces and electrostatic forces to interact with the functional groups of the proteins. This improves the proliferation and attachment of MC3T3-E1 cells on graphene oxide coated chitosan scaffolds [48,49,50]. The combination of graphene oxide nanocomposite with carboxymethyl chitosan scaffolds upgrades the osteogenic differentiation of seeded cells due to their high density of the functional groups. The carboxymethyl chitosan further enhances the cellular activity with the help of its amino and carboxyl groups [51]. Chitosan and graphene oxide that were incorporated with hyaluronic acid showed good attachment and spreading of MC3T3 cells. The proliferation of the cells increased by the addition of hyaluronic acid [52]. The reduced graphene oxide influences the stem cells function by nano topographic cues when combined with chitosan. The attachment of mesenchymal stem cells (MSC’s) on chitosan coated with reduced graphene oxide, increased in the initial days. After five days, the 5% concentration of reduced graphene oxide coated chitosan showed less cell proliferation when compared to the 0.05% and 0.5% reduced graphene oxide. This may be due to the cytotoxicity of reduced graphene oxide. Besides, the 5% reduced graphene oxide showed more calcium deposition and osteocalcin expression [53]. The combination of graphene oxide with bacterial cellulose increased the antimicrobial properties of the nanocomposites. The graphene oxide did not inhibit the proliferation of HEK293 cells and further decreased the bacterial attachment of Escherichia coli and Staphylococcus aureus by 95.6% and 65.35%, respectively [54]. Graphene oxide combined with chitosan and gelatin improves the biological properties of the scaffolds that were used for bone tissue engineering. The scaffolds were biocompatible and enhanced the proliferation of mouse mesenchymal stem cells into osteoblasts and increased the deposition of collagen in rat tibial bone defect [55].

Nair et al. [56] incorporated graphene oxide into gelatin/hydroxyapatite scaffolds to enhance the osteogenic differentiation of human mesenchymal stem cells. MSC’s on the scaffolds were cultured in two different culture conditions (media with and without osteogenic supplement) for quantitative evaluation of proliferation and osteogenic differentiation. There was no significant difference in cell proliferation between gelatin/hydroxyapatite scaffolds and graphene oxide/gelatin/hydroxyapatite scaffolds and between the culture conditions on all days. When compared to day 1, the number of cells were significantly higher on day 7 and day 14 in all groups. However, on day 21, the rate of cell proliferation was slightly reduced than day 14, still it was comparable to day 1 and day 7. Quantitative LDH activity measurement demonstrated that the percentage of viable cells from day 7 to 21 was equivalent and there was no considerable variation between the groups and time points. The results suggested that the cell proliferation on day 21 was reduced not due to the apoptosis/necrosis of cells. The graphene oxide scaffolds without the addition of osteogenic supplements, enhanced the proliferation and osteogenic differentiation of human mesenchymal stem cells [56]. Graphene oxide combined with silk fiber scaffolds are used for biomedical application and tissue engineering and the addition of graphene oxide has been shown to enhance the proliferation of MC3T3-E1 osteoblast cells when combined with silk fiber scaffolds. Furthermore, the graphene oxide reduced the pore diameter and eventually changed the pore morphology of the scaffolds [57]. Graphene oxide incorporated in starch nanocomposites has shown to improve the biological property [58]. The application of starch in tissue engineering, however, is minimal due to its brittleness and hydrophilicity.

### 3.2. Synthetic Biomaterials

Synthetic polymers are the commonly used materials for tissue regeneration due to their porosity, quicker degradation time, and superior mechanical strength. They have advantages over natural polymers, like longer shelf-life, cost effectiveness, ability to be tailored to obtain desired shape by milling and printing, can quickly be mass produced under controlled conditions, possess better cell differentiation properties, pore characteristics, and mechanical properties [59]. However, the drawbacks of synthetic biomaterials are that they lack cell adhesion sites and requires chemical modification to improve cell adhesion. The commonly used synthetic materials are Polylactic acid (PLA), Polyglycolic acid (PGA), Polycaprolactone (PCL), Poly lactide-co-glycolic acid (PLGA), calcium phosphate, hydroxyapatite (HA), β-Tricalcium phosphate (TCP), hydroxyapatite nanoparticles, glass-ceramics, bioactive ceramics, and bioactive glass [60,61]. These polymers have varying degrees of biocompatibility, biodegradability, and mechanical strength, but no single polymer possesses all these three essential properties at an optimal level. Hence, graphene derivatives are often integrated with synthetic biomaterials to improve the biomechanical behavior of the tissue engineering scaffolds.

#### Polymers/Graphene Derivatives Scaffolds in Bone Tissue Engineering

**Figure 2:**
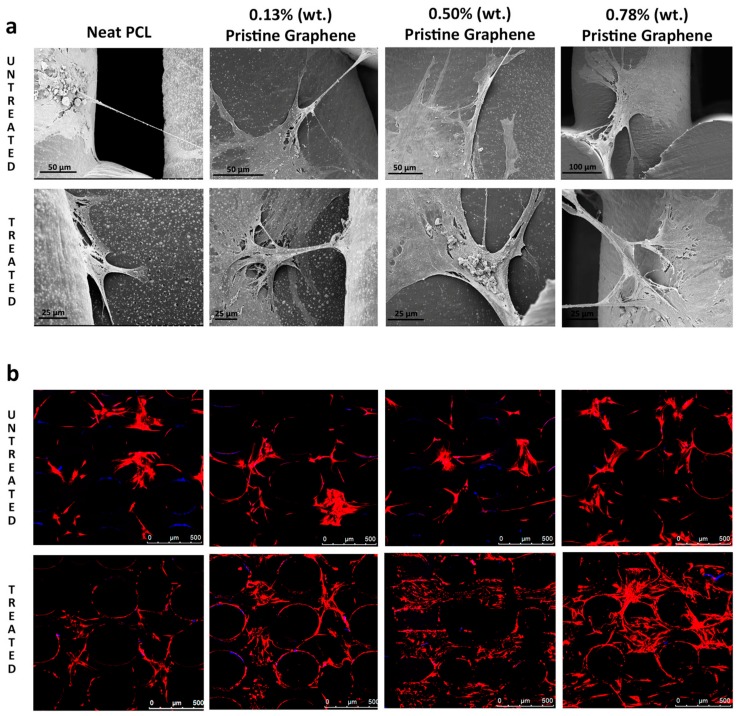
Proliferation and cell viability of human adipose derived stem cells on Polycaprolactone (PCL) and pristine graphene scaffolds (**a**) SEM images after 21 days culture; and, (**b**) confocal images after 28 days culture [64] (copyright 2016, Materials).

Polycaprolactone is a polymer with melting temperature of 60 °C and it is hydrophobic in nature [62]. Polycaprolactone has been wildly used in biomedical application for its excellent solubility and blend compatibility. Although Polycaprolactone has many advantages, owing to factors, such as slow degradation, resorption, and hydrophobicity, it might not be suitable to be used alone. Polycaprolactone can be three-dimensionally printed with graphene derivatives to enhance their osteogenic potential and biocompatibility. Graphene oxide has been shown to enhance the differentiation of mesenchymal stem cells and PC12 cells into osteo-and neuro-like cells. Song et al. [63] cultured mesenchymal stem cells and PC12 cells on the polycaprolactone/graphene oxide scaffolds and observed the cell growth and adherence. The cell proliferation on different scaffolds were analyzed using a CCK-8 assay after one, four, and seven days. Both the mMSCs and PC12-L cells grew well on different composite scaffolds with the extended culture time. The mMSCs proliferated slower on the PCL/GO composite scaffolds with 0.5- and 1.0-wt % GO. The PCL/GO scaffolds with 1.0-wt % GO exhibited significantly lower cell proliferation when compared with other scaffolds at the 4th day. The proliferation rates of mMSCs on the PCL/GO scaffolds with 0.3- and 0.1-wt % GO and the pure PCL scaffolds did not significantly differ. Similarly, PC12-L cells proliferated slower on the PCL/GO scaffolds with 0.5- and 1.0-wt % GO, and this difference was more prominent as the culture time was extended, especially on the scaffolds with 1.0-wt % GO. The results showed that 0.3% and 0.5% concentration of graphene oxide enhanced the differentiation of mesenchymal stem cells into osteogenic cells [63]. Wang et al. [64] stated that the addition of pristine graphene enhances the proliferation and cell viability of human Adipose derived stem cells. The polycaprolactone and pristine graphene scaffolds were treated with sodium hydroxide (NaOH) and the results showed that the scaffolds containing pristine graphene treated with NaOH exhibited an increase in fluorescence intensity when assessed using Almar Blue Assay. For the scaffolds that were treated with NaOH, the addition of pristine graphene increased the cell viability and proliferation. Pristine graphene scaffolds showed better biological performance than the control polycaprolactone scaffolds. They further assessed the cell attachment and morphology of the scaffolds by SEM and laser confocal microscopy. The addition of pristine graphene showed extensive cell attachment and spreading. The confocal images of the scaffolds containing pristine graphene showed that larger number of cells were present, morphology of the cells were maintained, and the cells showed high confluency [64] (Figure 2).

**Figure 3:**
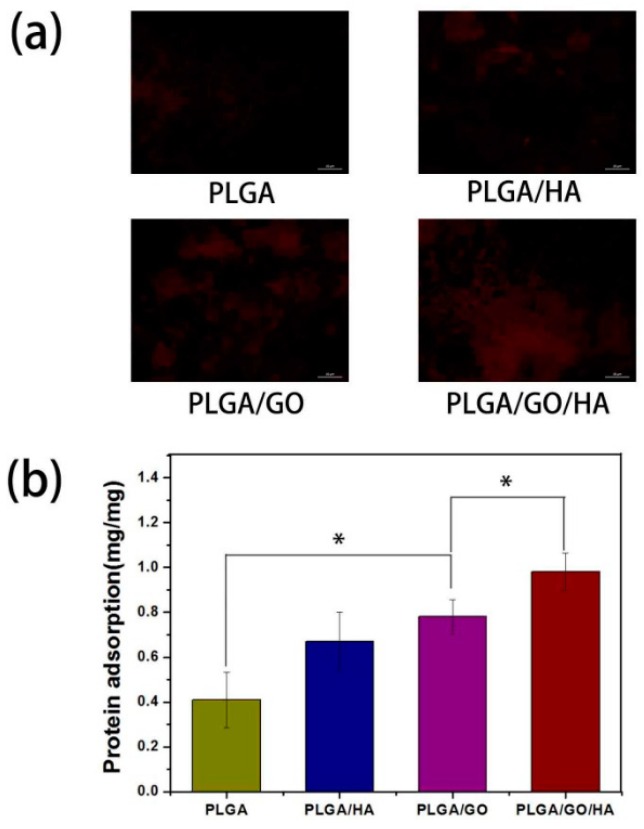
Protein adsorption and cell adhesion of Poly lactide-co-glycolic acid (PLGA), Poly lactide-co-glycolic acid/hydroxyapatite (PLGA/HA), PLGA/graphene oxide (GO), and PLGA/GO/HA nanofibrous matrices. (**a**) Fluorescence images of the Rhodamine B labelled BSA adsorption on PLGA, PLGA/HA PLGA/GO and PLGA/GO/HA; and, (**b**) The adsorption of protein onto the PLGA, PLGA/HA, PLGA/GO, and PLGA/GO/HA nanofibrous matrices. PLGA/GO/HA nanofibrous shows increase protein adsorption (n = 5; * indicates *p* < 0.05) [70] (Copyright 2017, PLoS ONE).

Polyvinyl alcohol is another synthetic biodegradable polymer that is soluble in water and hydrophilic in nature. Poly vinyl alcohol (PVA) is used in biomedical application combined with graphene oxide in the form of three-dimensionally printed scaffolds [65,66]. Polyvinyl alcohol and graphene oxide scaffolds are used for bone tissue engineering. Shuai et al. [67] produced interconnected porous nanocomposite by laser sintering. The graphene oxide added enhanced the proliferation and differentiation of osteoblast like cells and the addition of 2.5 wt % of graphene oxide increased the strength of the scaffolds [67]. Iontia et al. [68] added gelatin to the PVA/GO scaffolds and found that MC3T3-E1 preosteoblast and murine cells were cytocompatible [68]. Qi et al. [69] prepared nanocomposite PVA/GO scaffolds while using electrospinning. There was an increase in cell attachment and proliferation of mouse osteoblast MC3T3-E1 cells. The addition of graphene oxide did not affect the growth and proliferation of the cells. The cells showed a wide spread distribution on to the scaffolds [69]. Fu et al. [70] stated that the incorporation of graphene oxide into PLGA/HA nano fibres scaffolds enhanced the cell proliferation and osteogenic differentiation. The graphene oxide increased the protein absorption rate and hydrophilicity of the scaffolds significantly. The adsorption of proteins onto the surface of materials is highly related to biocompatibility of the materials. Bovine serum albumin (BSA) was selected as a model protein to examine the adsorption efficiencies of synthesized nanofibrous matrices. The BSA adsorption was determined to be 0.67 ± 0.13 and 0.78 ± 0.13 mg on PLGA/HA and PLGA/GO, respectively, which were obviously higher than that on PLGA nanofibrous matrices. The higher protein adsorption capacities of nanofibrous matrices might result from their improved surface properties and larger specific surface areas after the incorporation of HA and GO. The highest protein adsorption was obtained for the PLGA/GO/HA nanofibrous matrices, nearly 1.46 and 1.25 times of adsorption rates than those of PLGA/HA and PLGA/GO nanofibrous matrices, respectively (Figure 3). The PLGA/GO/HA scaffolds showed an increase in cell adhesion and proliferation of MC3T3-E1 cells, as well as an increase in ALP activity, mineral deposition, and osteogenic related gene expression [70].

**Figure 4:**
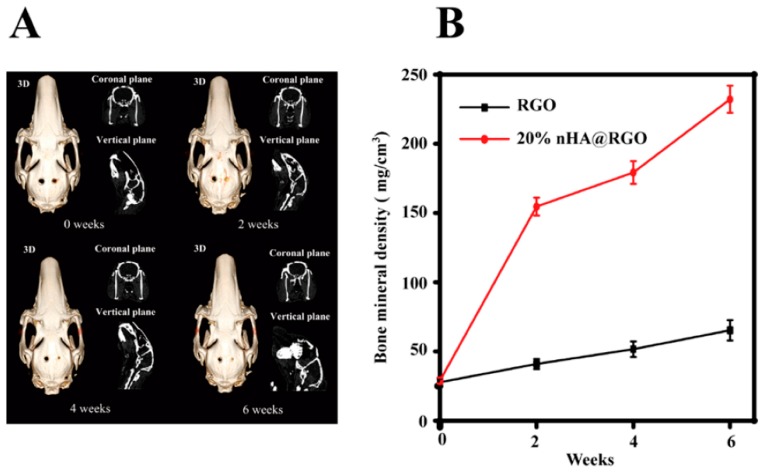
Proliferation and of bone mineral density (BMD) of rat bone mesenchymal cells. (**A**) Computed tomography analysis of the defect repair at different time interval. The left defect planted by free reduced graphene oxide scaffold, and the right treated with 20% nHA@RGO scaffold. (**B**) The change of bone mineral density (BMD) after the scaffold implantation [71] (copyright 2017, Carbon).

**Figure 5:**
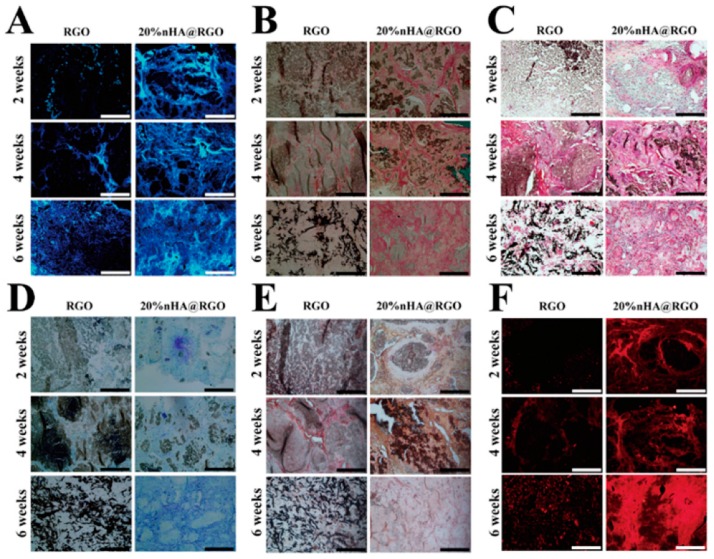
Proliferation, osteogenic gene expression and ALP activity of rat bone mesenchymal cells (**A**) DAPI; (**B**) Goldner’s; (**C**) Masson’s trichrome staining; (**D**) Toluidine Blue; (**E**) alkaline phosphatase (ALP); (**F**) OCN [71] (copyright 2017, Carbon).

**Figure 6:**
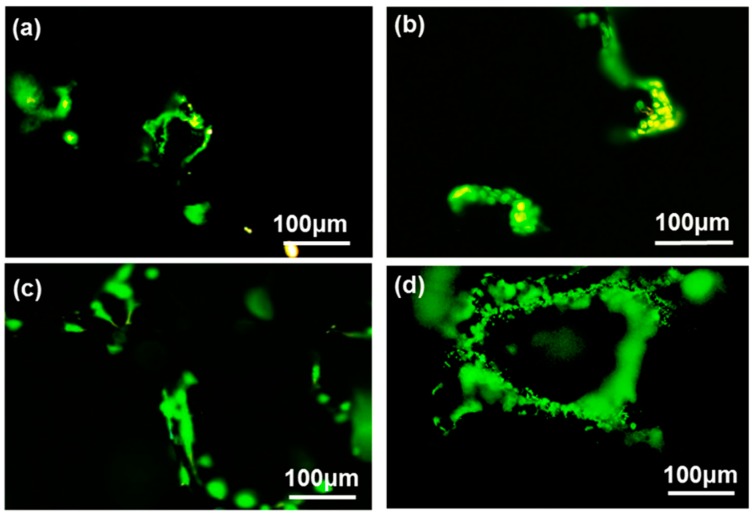
Cytotoxicity of graphene-hydroxyapatite hybrid (GHB) scaffold on MC3T3-E1 cells. (**a**,**b**) second and fourth day(3D Graphene Foam) (**c**,**d**) 2nd and 4th day (3D GHBs), respectively [72] (copyright 2018, Crystals).

Wei et al. [71] produced a three-dimensional porous scaffold containing reduced graphene oxide and nano hydroxyapatites for bone tissue engineering. The in vitro evaluation showed that the scaffolds enhanced the proliferation, osteogenic gene expression of rat bone mesenchymal cells, and increased the ALP activity. The scaffolds placed in the calvarial defects of rabbits showed good bone healing in six weeks after implantation and there was increased proliferation and improved collagen deposition when observed by computed tomography and histological analysis [71] (Figure 4 and Figure 5). Wei et al. [72] produced a three-dimensional graphene-hydroxyapatite hybrid (GHB) scaffold with calcium phosphate salt that was electrochemically deposited on to the graphene foam framework. Voltage and temperature were used to control the morphology of HA deposited. The cell culture of MC3T3-E1 osteoblast cells on GHB scaffolds showed good biocompatibility and increased proliferation. The cytotoxicity and health status of the cells were evaluated by Acridine orange-ethidium bromide double staining assay. Both the three-dimensional graphene foams and GHB scaffolds showed higher densities of cells and were confirmed by green fluorescence indicating live cells. The dead cells were identified by orange color. Both the scaffold groups didn’t show any dead cells (Figure 6). The cells were co-cultured on second day and fourth day. The outcomes of the study showed that MC3T3-E1 osteoblast cells could grow, adhere, and proliferate on GHB scaffolds. There was an increase in ALP activity, which showed that GHB scaffolds enhanced the differentiation of osteoblast cells to mature osteoblasts [72].

Ali et al. [73] prepared a novel graphene oxide incorporated silicate-doped nano hydroxyapatite composite scaffold for bone tissue engineering. Electrospun PCL scaffolds were fabricated and the Graphene oxide-Hydroxyapatites nano composite were reinforced within the scaffolds. Graphene oxide was added in the concentration of 2 wt % and 4 wt %. The scaffolds were tested for protein absorption and desorption while using FBS solution. The results showed that the graphene oxide enhanced the absorption and decreased the desorption of proteins. The scaffolds were biocompatible when treated with human osteosarcoma cell lines. The scaffolds showed good adhesion, proliferation of cell lines, and increased ALP activity [73].

**Figure 7:**
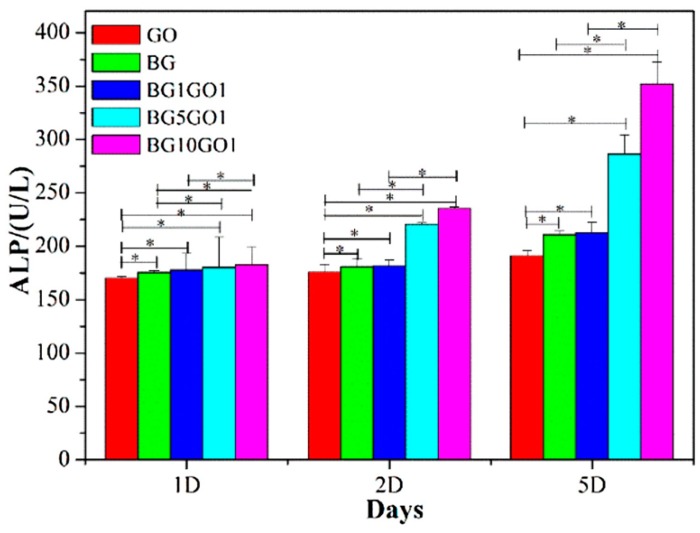
Alkaline phosphatase activity (Error bars indicate SD, n = 6. * indicates *p* < 0.05) [79] (copyright 2016, Colloids Surf. B: Biointerface).

Bioactive glass has been widely used in the field of bone tissue engineering because of its ability to form bioactive hydroxyapatite layer, thereby forming a strong bond between implant and the surrounding bone [74,75]. The bioactive glass does not have sufficient mechanical property to replace the bone tissues and needs either organic or inorganic materials to be added along to improve their mechanical strength. Graphene has been shown to have excellent mechanical strength with the added advantage of good biocompatibility and it does not cause any inflammatory changes to the surrounding microenvironment [76,77,78]. Fan et al. [79] produced a scaffold by combining graphene nanosheet with bioactive glass to improve the mechanical strength of bioactive glass (BG’s). The addition of graphene nanosheets increased the mechanical strength of the scaffolds, furthermore, the graphene nanosheets scaffolds showed excellent biocompatibility and cell proliferation. The osteoblast cells adhered and were wide spread on the scaffolds containing graphene nanosheets. Moreover, an increase in the proliferation of the osteoblast cells and ALP activity established the osteogenic potential of the graphene nanosheet coating onto the scaffolds. Alkaline phosphatase activity was observed after the cells were incubated on the surface of GO, BGs, BG1GO1, BG5GO1, and BG10GO1 for one, two, and five days, respectively. On one day, ALP activity no apparent difference was observed between GO, BGs, BG1GO1, BG5GO1, and BG10GO1. As time progressed, an increased trend of ALP activity was observed, and the ALP activity of BG10GO1 was higher than GO, BGs, BG1GO1, and BG5GO1 on two and five days, indicating the highest osteogenic differentiation activity of the MC3T3-E1 cells on the surface of BG10GO1 [79] (Figure 7).

Cheng et al. [78] combined graphene with nano-58S bioactive glass for bone tissue engineering application. The scaffolds were fabricated using selective laser sintering. The results of the study showed that graphene potentiated the mechanical strength of the scaffolds. The cell culture work was done by immersing the scaffolds in SBF solution for seven days that resulted in well-formed HCA layer and the calcium/phosphorus ratio was 1.69. Cell culture studies further showed that the human MG63 cells adhered and grew on the surfaces of the scaffolds containing graphene. Graphene promoted the increase in proliferation of cells [78]. Turk et al. [80] stated that graphene containing PCL coated with borate based 13–93 BC bioactive material, increased the electrical conductivity of the scaffolds. Graphene containing scaffolds were biocompatible with large amount of viable MC3T3-E1 preosteoblast cells after incubation for seven days by XTT test. The cell viability started to decrease after 14 days of incubation. The graphene-based scaffolds also demonstrated an increase in ALP activity, which accounted for their osteoblastic differentiation. When subjected to incubation for three days, a wide spread of MC3T3-E1 cells on the scaffolds coated with graphene were observed and the cells exhibited flat appearance [80].

## 4. Role of Graphene in Stem Cell Proliferation

Graphene, a two-dimensional (2D) nanomaterial has set an enormous trend in research and development in the burgeoning field of biomedical application and bone tissue engineering. Despite their potentiality, very few researchers have attempted to use graphene in human stem cell research. Most of the studies that were done with graphene had focused on increasing the proliferation, adhesion, and growth of stem cells. There has to be a controlled proliferation and differentiation of the stem cells in order to attain successful bone regeneration. This can be achieved with the help of growth factors and osteogenic inducers [81]. Human bone marrow derived stem cells can be allowed to differentiate and proliferate in a controlled manner by combining them with artificial scaffolds to regenerate the lost bone tissues [81,82,83,84,85]. Graphene derivatives have been shown to support stem cell attachment and differentiation, and are also used for various bone tissue regeneration [86]. The physical, chemical, and mechanical properties of graphene allows for the stem cells to differentiate and proliferate on to the scaffolds [87]. Although graphene has been researched for implementation in stem cell research, the exact interaction between graphene derivatives and the stem cells is still unclear. Graphene oxide has been shown to enhance the osteogenic potential of stem cells. Wu et al. [35] combined graphene oxide with beta tricalcium phosphate bio ceramics and evaluated the osteogenic potential of the scaffolds. The scaffolds containing graphene oxide enhanced proliferation, osteogenic gene expression, and ALP activity of human bone marrow stem cells. The scaffolds without graphene oxide (as control) showed less osteogenic differentiation compared to the scaffolds with graphene oxide. The in vivo study showed that β-TCP-GRA scaffolds had greater de novo bone formation in the calvarial defects both at four and eight weeks post implantation when compared with β-TCP scaffolds. Micro-CT analysis showed significantly more new bone formation in the β-TCP-GRA group compared with the β-TCP group. The BV/TV ratio of the β-TCP-GRA group (26.12 ± 4.44% and 44.83 ± 10.82%) was significantly higher as compared with β-TCP (16.64 ± 4.57% and 30.41 ± 4.10%) at weeks 4 and 8, respectively. The exact mechanism accounting for an increase in osteogenic potential of graphene oxide is by activation of Wnt/b-catenin signaling pathway in hBMSCS’s [35]. Akhavan et al. [88] showed that graphene nano grids enhanced the osteogenic capacity of human mesenchymal stem cells (hMSC’s). The growth and shape of the stem cells were not affected by the incorporation of graphene, suggesting the excellent biocompatibility of graphene [88]. Graphene has been shown to accelerate the growth and differentiation of human mesenchymal stem cells similar to the differentiation influenced by bone morphogenic protein (BMP-2) [32]. Three-dimensionally printed graphene foams have been shown to promote osteogenic differentiation and maintain the viability of human mesenchymal stem cells [89].

Luo et al. [90] stated that PLGA/GO enhanced the osteogenic differentiation of human mesenchymal stem cells. Graphene oxide incorporated on electrospun poly (lactic-co-glycolic acid) scaffolds helped in the adhesion and proliferation of hMSC’s, thereby accelerating the osteogenic potential when compared to the scaffolds without graphene oxide (PLGA-control). Additionally, graphene oxide enhanced the hydrophilic performance and absorption ability of PLGA nanofibers [90]. Tatavarty et al. [91] stated that graphene oxide shows a synergetic effect when combined with osteoinductive material. Graphene oxide with calcium phosphate nano composite have shown synergetic effects on the osteogenic differentiation of human mesenchymal stem cells and exhibited superior stiffness when compared to graphene oxide or calcium phosphate alone. Such an increase in material stiffness could induce mechanotransduction effect, which regulates stem cell differentiation. The combination of graphene oxide with calcium phosphate nano composite showed increased bone nodule formation when compared to the calcium phosphate or graphene oxide alone, which demonstrates the synergetic effect of graphene oxide combined with osteoinductive or osteoconductive materials [91]. Graphene that is used for osteogenic differentiation is summarized in (Table 1).

## 5. Future Perspectives and Conclusions

There are various factors to be considered in designing a scaffold for bone tissue engineering. Although some of the biomaterials that were previously used as a scaffold in clinical trials are available in the commercial market, the methodology and their applications are not always well-balanced. Graphene with its unique properties are potentially validated to be used as bone tissue engineering scaffolds. Research on the contribution of graphene derivatives to bone tissue engineering is still modern, and various studies are ongoing to understand them better. Numerous studies in stem cell research are being conducted in order to explore graphene for its osteogenic potential, and proliferation and differentiation of stem cells. Despite its advantages, graphene derivatives are subjected to few challenges. The toxicity of graphene at higher concentrations and non-biodegradable nature needs further investigations. The interaction of graphene with the biologic cells is yet to be further evaluated in depth. Literature should furnish a wide range of comparative studies in the future, between graphene and the other biomaterials before commercializing them in the market. Concurrently, many in vivo studies involving graphene needs to be tested to understand the exact metabolic pathways in which graphene interacts with the microenvironment and bone tissue regeneration. The success of bone regeneration depends on the proliferation of stem cells. The availability and affordability of graphene derivatives has led to the outcome of many researchers conducting studies to validate the proliferation and differentiation of stem cells by the incorporation of graphene derivatives. A remarkable aspect of graphene derivatives is that their chemical nature seems to act on the proliferation of stem cells, raising the possibility of favoring this process by modifying the chemical configuration. Cell-based bone tissue engineering requires a cell component from the appropriate origin that could be able to repair the target tissues and also a biocompatible material that provides a three-dimensional scaffold for the chosen cells. The ideal combination of cell and scaffold sources varies in relation to the target tissue to be created. Moreover, there have been recent advances in biomaterial synthesis and fabrication tools resulting in the creation of functional and bioactive scaffolds that stimulate different cell functions. Titanium and synthetic biomaterials are used for the reconstruction of lost bone tissues. Titanium is considered as a best suitable material for bone regeneration due to its mechanical strength and biocompatibility. Although titanium helps in better osseointegration, there are chances of development of fibrous tissues, which may lead to failure of implants. To overcome this drawback, graphene can be coated onto the surface of implants to enhance better osseointegration and bone formation. Although graphene enhances the bone formation, the long term success of graphene coated on to the implant surface is yet to be researched. Attempts had been made to use Graphene as a carrier for delivery of BMP-2 for better osseointegration. Nonetheless, the outcomes in clinical application are questionable. Graphene derivatives have been tried along with synthetic biomaterials and titanium for modular endoprosthesis in reconstruction of fractured and resected bone segments. Perhaps, studies are still in the early stages of in vitro analysis and in vivo experiments are yet to be conducted. Prospective in vitro and in vivo studies can be helpful to elucidate the full mechanisms of action of the actual available configurations of graphene-based scaffolds. Indeed, the foreseeable future can take advantage of this carbon material to be used as a promising scaffold for bone tissue engineering.

## Figures and Tables

**Table 1:** Osteogenic potential of graphene in bone tissue engineering scaffolds.

Material	Analysis	Outcomes	Reference
rGO-Chitosan	SEM, Alizarin Red staining, and immunofluorescence	The differentiation on rGO-chitosan substrate was higher than the ones obtained on the chitosan Substrate and polystyrene regardless of the use of osteogenic induction media.	[53]
rGO-PEDOT	Immunofluorescence staining, Alizarin Red S staining	The multifunctional rGO-PEDOT bioelectronic interface was used for manipulating attachment and orientation of MSC. The device acted as a drug releasing model under electrical modulation.	[92]
GO	Immunofluorescence, microcomputed tomography, and Goldner trichrome	The osteogenetic differentiation of human BMMSCs on Ti/GO substrate was higher compared to Ti substrate.	[31]
GONR, rGONR	Immunofluorescence staining and Alizarin Red staining	Graphene nanogrids increase the osteogenic differentiation of BMSC; the differentiation coincides with the patterns of the nanogrids.	[88]
CVD	Immunofluorescence staining	The cells adhered and proliferated more on CVD-grown graphene than on SiO_2_ substrates.	[93]
CVD, GO	Immunofluorescence staining and Alizarin Red staining	Graphene was capable of preconcentrating osteogenic differentiation factors. GO strongly enhances adipogenic differentiation.	[87]
CVD	Cell viability assay, immunofluorescence staining, and Alizarin Red staining	CVD-grown graphene allowed the proliferation of MSC and increased the differentiation towards osteoblast.	[32]
3DGp	Immunofluorescence staining and SEM	3DGp maintains MSC viability and promotes osteogenic differentiation without the use of chemical inducers.	[89]
CaS-G	MTT, SEM, and RT-PCR	Cell adhesion was enhanced by adding 1.5% of graphene to the material as compared to the calcium silicate alone.	[94]
SGH	MTT, H & E, immunofluorescence staining, and Alizarin staining	The self-supporting graphene hydrogel (SGH) film allows cell adhesion and proliferation and accelerates the osteogenic differentiation without chemical inducer.	[95]
GO-CaP	Alizarin Red S staining RT PCR and immunofluorescence	The GO-CaP nanocomposite exhibited superior osteoinductivity compared to individual or combined effects of GO and CaP.	[91]
Carbon nanotubes and graphene	SEM, Elisa, and H & E staining	Cells in PLLA composite scaffolds containing 3 wt % of graphene presented higher expression of osteogenesis-related proteins, calcium deposition, and the formation of type I collagen.	[96]
Graphene hydrogel	MTT and SEM	Graphene 3D hydrogel allows cell proliferation and attachment confirming the biocompatibility of the graphene hydrogel scaffolds.	[97]

Source: Reference [98] (copyright 2015, Stem Cells International).

## Abbreviation

MSC’s	human mesenchymal stem cells
BMSCs	Bone marrow-derived mesenchymal stem cells
PLA	Polylactic acid
PGA	Polyglycolic acid
PCL	Polycaprolactone
PLGA	Poly lactide-co-glycolic acid
HA	hydroxyapatite
TCP	β-Tricalcium phosphate
NaOH	sodium hydroxide
PVA	Polyvinyl alcohol
GO	graphene oxide
GHB	graphene-hydroxyapatite hybrid
ALP	Alkaline phosphatase
SBF	simulated body fluid
